# Construction of a prognostic signature based on T-helper 17 cells differentiation–related genes for predicting survival and tumor microenvironment in head and neck squamous cell carcinoma

**DOI:** 10.1097/MD.0000000000041273

**Published:** 2025-01-24

**Authors:** Shiqin Chen, Pingcun Wei, Gang Wang, Fan Wu, Jianjun Zou

**Affiliations:** a Department of Otorhinolaryngology and Head and Neck Surgery, Anhui No.2 Provincial People’s Hospital, Hefei, Anhui, China; b Department of Otolaryngology, Hangzhou Red Cross Hospital (Zhejiang Hospital of Integrated Traditional Chinese and Western Medicine), Hangzhou, Zhejiang, China.

**Keywords:** consensus clustering, head and neck squamous cell carcinoma, machine learning, nomogram, T-helper 17 cells

## Abstract

T-helper 17 (Th17) cells significantly influence the onset and advancement of malignancies. This study endeavor focused on delineating molecular classifications and developing a prognostic signature grounded in Th17 cell differentiation–related genes (TCDRGs) using machine learning algorithms in head and neck squamous cell carcinoma (HNSCC). A consensus clustering approach was applied to The Cancer Genome Atlas-HNSCC cohort based on TCDRGs, followed by an examination of differential gene expression using the limma package. Machine learning techniques were utilized for feature selection and model construction, with validation performed using the GSE41613 cohort. The interplay between the predictive marker, immune landscape, immunotherapy response, drug sensitivity, and clinical outcomes was assessed, and a nomogram was constructed. Functional evaluations of TCDRGs were conducted through colony formation, transwell invasion, and wound healing assays. Two distinct HNSCC subtypes with significant differences in prognosis were identified based on 87 TCDRGs, indicating different levels of Th17 cell differentiation. Thirteen differentially expressed TCDRGs were selected and used to create a risk signature, T17I, using the random survival forest algorithm. This signature was associated with grade, chemotherapy, radiotherapy, T stage, and somatic mutations. It was revealed that there were differences in the immune response–related pathways between the high- and low-risk groups. Inflammatory pathways were significantly activated in the low-risk group. The T17I signature was associated with immune infiltration. Specifically, there was a higher infiltration of immune activation cells in the low-risk group, whereas the high-risk group had a higher infiltration of M2 macrophages. In addition, the T17I signature was significantly associated with drug sensitivity. A nomogram combining age, radiotherapy, and the T17I signature accurately predicted the prognosis of patients with HNSCC. Finally, in vitro experiments confirmed that knockdown of *LAT* gene expression promotes proliferation, metastasis, and invasion of HNSCC cells. In conclusion, this study successfully identified molecular subtypes and constructed a prognostic signature and nomogram based on TCDRGs in HNSCC, which may aid in personalized treatment strategies.

## 1. Introduction

Head and neck squamous cell carcinoma (HNSCC), which stems from the mucosal epithelial cells of the oral cavity, pharynx, and larynx, is among the most prevalent malignancies in the head and neck area. HNSCC imposes a significant global health burden. In 2020, more than 800,000 new cases were reported, and around 400,000 deaths occurred.^[1]^ The World Health Organization projects that by 2030, an estimated 439,000 new cases of mouth and oropharyngeal cancer will be present.^[2]^ Unfortunately, due to nonspecific early symptoms, many patients are diagnosed at advanced stages.^[3]^ HNSCC has a relatively high recurrence rate and distant metastasis rate, with a 5-year survival rate below 65%.^[4]^ Although recent advancements in tumor immunotherapy, such as the approval of pembrolizumab and nivolumab as first-line treatments for recurrent or metastatic HNSCC, have offered some hope, the majority of patients with HNSCC show limited clinical responses to immune checkpoint inhibitors.^[5,6]^ This highlights the significant challenges in HNSCC research, including the heterogeneity of the disease, the presence of multiple genetic and epigenetic alterations, and the complex interplay between the tumor microenvironment (TME) and immune system. These factors contribute to the variability in treatment responses and the development of resistance to therapy. Thus, elucidating the molecular characteristics and prognostic markers of HNSCC is crucial for improving therapeutic efficacy and patient survival rates.^[7–10]^

T-helper 17 (Th17) cells, a unique subset of helper T cells, play a pivotal role in maintaining homeostasis and regulating autoimmune diseases and cancer progression.^[11,12]^ Recent studies have shown that Th17 cells participate in inflammation and autoimmunity and also exhibit complex roles within the TME.^[13]^ On one hand, Th17 cells and related cytokines, such as interleukin-17 (IL-17), can promote tumor growth and progression and on the other hand, they can enhance antitumor immune responses, particularly in conjunction with CD8^+^ T cells.^[14]^ Furthermore, the balance between Th17 cells and regulatory T cells is considered essential for maintaining immune homeostasis.^[15]^ In the field of cancer immunotherapy, Th17 cells are of particular interest due to their potential immunomodulatory capabilities. For example, recent studies have shown that, under certain conditions, Th17 cells can synergize with CD8^+^ T cells to facilitate tumor regression.^[16–18]^ However, the mechanisms governing Th17 cell functions remain complex and require further investigation to clarify their precise roles in tumor development.

**Figure 1.**
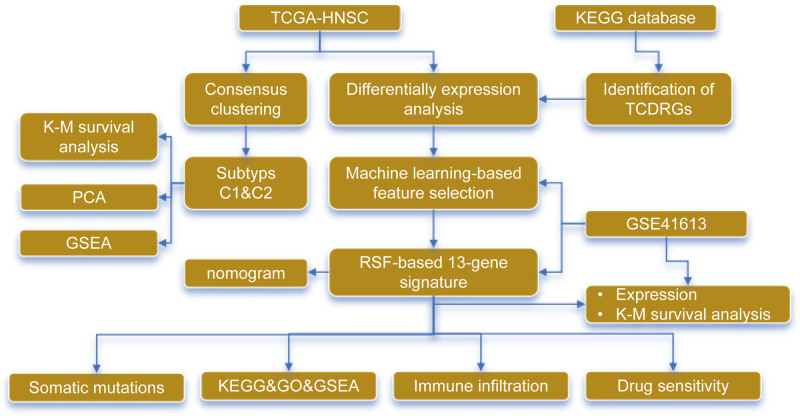
Workflow diagram of the study. GO = Gene Ontology, GSEA = gene set enrichment analysis, HNSCC = head and neck squamous cell, KEGG = Kyoto Encyclopedia of Genes and Genomes, K–M = Kaplan–Meier, PCA = principal component analysis, RSF = random survival forest, TCDRG = T-helper 17 cell differentiation–related genes, TCGA = The Cancer Genome Atlas.

Given the challenges in HNSCC research and the limited efficacy of current treatments, in this study, we explored the expression, mutation, and prognostic relevance of Th17 cell differentiation-related genes (TCDRGs) in HNSCC through bioinformatics analysis. Using innovative machine learning algorithms, we constructed a prognostic signature and nomogram based on TCDRGs, elucidating the relationships between TCDRGs and the tumor immune landscape, immunotherapy response, and drug sensitivity in HNSCC. This work lays the foundation for a deeper understanding of the biological functions of TCDRGs in HNSCC. Figure 1 illustrates the workflow of the study.

## 2. Materials and methods

### 2.1. Data acquisition and processing

For The Cancer Genome Atlas (TCGA)-HNSCC project, transcriptomic data, somatic mutations, and clinical–pathological information were sourced from TCGA (https://portal.gdc.cancer.gov/). Samples lacking complete clinical–pathological data were removed, leading to a final group of 432 patients. The GSE41613 cohort, which has 95 cases, had its transcriptomic and survival data downloaded from the Gene Expression Omnibus (https://www.ncbi.nlm.nih.gov/). The limma package was utilized to normalize the transcriptomic data and then log2 transform it. The sva package was applied to correct batch effects. Table 1 presents the clinicopathologic features of the cohorts. TCDRGs were retrieved from the Kyoto Encyclopedia of Genes and Genomes (KEGG, https://www.kegg.jp/) (Table S1, Supplemental Digital Content, http://links.lww.com/MD/O281).

**Table 1:** Clinical and pathological characteristics of TCGA-HNSCC and GSE41613 cohorts.

Characteristics	TCGA-HNSCC	GSE41613
Cases	432	95
Outcome		
Dead	155	50
Alive	277	45
Age		
<60	195	49
≥60	237	46
Gender		
Female	111	30
Male	321	65
T stage		
T1	27	
T2	129	
T3	114	
T4	162	
N stage		
N0	214	
N1	64	
N2	138	
N3	16	
M stage		
M0	418	
M1	14	
Clinical stage		
Stage I/II	100	40
Stage III/IV	332	55
Source tissue		
Oral cavity	108	
Pharynx	49	
Lip	66	
Mandible	1	
Larynx	100	
Tongue	108	

HNSCC = head and neck squamous cell carcinoma, TCGA = The Cancer Genome Atlas.

### 2.2. Consensus clustering analysis

Consensus clustering was performed using the ConsensusClusterPlus package. The TCDRGs dataset from the TCGA-HNSCC cohort was analyzed, setting the maximum number of clusters to 6, the number of subsamples to 50, and the clustering algorithm to partition around medoids. Default parameters were used for other settings. Molecular subtyping was conducted, and then Kaplan–Meier (K–M) survival analysis was performed to assess the prognostic differences among subtypes. In addition, principal component analysis and gene set enrichment analysis (GSEA) were also carried out.

### 2.3. Construction of prognostic features

Ten machine learning algorithms, including support vector machine, least absolute shrinkage and selection operator, gradient boosting machine, random forest, elastic net, stepwise Cox, ridge, CoxBoost, super partial correlation, and partial least squares with Cox regression, were employed for feature selection and model building. An analysis of differential expression was carried out to identify genes with varying expression levels. A sequential approach was used: differential expression analysis was conducted to pinpoint genes with altered expression; 101 different algorithm combinations were applied to the TCGA-prostate adenocarcinoma cohort using a leave-one-out cross-validation framework to fit a predictive model; all models were tested in the GSE41613 cohort; the model with the highest mean Harrell concordance index (C-index) was selected as the optimal model. Based on the median score, participants were divided into 2 categories: high risk and low risk. To evaluate the survival outcomes, K–M curves were plotted, and the area under the receiver operating characteristic (ROC) curve (AUC) was calculated. The statistical significance of the difference in survival rates between these 2 groups was determined using the log-rank test.

### 2.4. Enrichment analyses

Functional annotation and pathway analysis were conducted using the clusterProfiler package, focusing on Gene Ontology (GO) and KEGG pathways. Differentially expressed genes between the high- and low-risk groups were identified with the limma package, applying a significance threshold of *P* value <.05 and an absolute log2 fold change >1. After converting gene IDs, enrichment analysis was performed using the enrichKEGG and enrichGO functions. Dot plots were generated to illustrate selected results. Hallmark gene sets from the Molecular Signatures Database were retrieved, and GSEA was conducted using the differential expression data. The 10 most significantly enriched pathways were visualized using the gseaplot2 function.

### 2.5. Somatic mutation analysis

The somatic mutation data from the TCGA-HNSCC cohort were examined using the maftools package. Oncoplots were generated to display the 10 most frequently mutated genes in both the high- and low-risk groups. Tumor mutational burden (TMB) was quantified, and the differences in TMB between the high- and low-risk groups were evaluated using the Wilcoxon rank-sum test. The relationship between TMB and prognostic factors was assessed through Pearson correlation analysis, and the results were presented in a scatter plot.

### 2.6. Assessment of the tumor immune microenvironment

The tumor immune microenvironment was characterized using the IBOR package. Cell-type Identification by Estimating Relative Subsets of RNA Transcripts (CIBERSORT) was employed to estimate the immunoinfiltration conditions within the TCGA-HNSCC cohort, and the Wilcoxon rank-sum test was used to compare the differences between groups. Pearson correlation analysis was conducted to explore the relationship between TCDRGs and immune cell infiltration. The Estimation of STromal and Immune cells in MAlignant Tumor tissues using Expression data (ESTIMATE) algorithm was utilized to determine the immune score and tumor purity, while the immunophenoscore algorithm was used to calculate the immunophenoscore. The tumor immune dysfunction and exclusion (TIDE, http://tide.dfci.harvard.edu/) algorithm was applied to evaluate the potential response to immune therapy.

### 2.7. Drug sensitivity analysis

Drug sensitivity for the TCGA-HNSCC cohort was predicted using the pRRophetic package. The Wilcoxon rank-sum test was employed to compare sensitivity differences between groups. To assess the correlation between sensitivity and TCDRGs, Pearson correlation analysis was carried out.

### 2.8. Construction and performance evaluation of nomograms

To identify independent prognostic factors in the TCGA-HNSCC cohort, univariate and multivariate Cox regression analyses were carried out, with factors having *P* values <.05 being selected. Using the rms package, nomograms were constructed and evaluated. The predictive performance of the nomogram was assessed by means of calibration curves, ROC curves, and decision curves.

### 2.9. Cell culture and transfection

The SCC-4 and UM-SCC-47 cells were cultured in 1640 medium (Gibco, Grand Island) supplemented with 10% fetal bovine serum (FBS) at 37°C in a 5% CO_2_ incubator (Nikon, Japan, Tokyo). Only cells in the logarithmic growth phase and free from contamination were used for experiments. For small interfering RNA (siRNA) transfection, cells were grown to subconfluence and then transfected with 50 nM siRNA targeting *LAT* or nonsilencing control siRNA (Guangzhou RiboBio Co., Ltd., Guangzhou, China) using Lipofectamine RNAiMAX transfection reagent (Thermo Fisher Scientific, Waltham). Cells were harvested 48 hours posttransfection for further experimentation.

### 2.10. Western blot analysis

Posttransfection, cell cultures were harvested thrice with ice-cold phosphate buffered saline (PBS) and processed further. To each well (150 μL), we added lysis buffer (Beyotime, Jiangsu, China) and prepared cell lysates via sonication. Centrifugation was performed at 13,000 rpm for 10 minutes at 4°C, followed by quantification of the total protein content using a bicinchoninic acid protein assay kit (Beyotime). Before immunoblotting, 40 μg of proteins were loaded onto gels. After blot transfer, polyvinylidene fluoride membranes were rinsed with Tris-buffered saline to eliminate the transfer buffer. Membranes were then incubated with 3% bovine serum albumin at ambient temperature on a shaker for 1 hour, followed by overnight incubation at 4°C with primary antibodies targeting *LAT* and GAPDH under agitation. Primary antibodies were diluted in 3% bovine serum albumin containing NaN_3_ preservative (1:2000 for housekeeping genes; 1:1000 for other proteins). After overnight incubation, membranes were washed and primary antibodies were retrieved if necessary. After incubation, polyvinylidene fluoride membranes were rinsed 3 times with 1× Tris-buffered saline with Tween-20 for 10 minutes each, and the protein bands were detected using an electrochemiluminescence system.

### 2.11. RNA extraction and quantitative real-time polymerase chain reaction

Total RNA extraction occurred 48 hours after transfection via the Trizol technique (Invitrogen, Carlsbad, California), which was followed by complementary DNA synthesis employing real-time polymerase chain reaction (RT-PCR) with SYBR Green reagents (Thermo Fisher Scientific). Amplification was performed using the PCR-7500 Real-Time PCR System (Applied Biosystems, Shanghai, China), and internal controls were normalized to GAPDH. Fold change quantifications were computed using the 2^−ΔΔCq^ approach. Primer details can be found in Table 2.

**Table 2:** Primers used in qRT-PCR assay.

Genes	Direction Direction	Sequence (5′–>3′)
*Lat*	Forward primer	GATGAGGACGACTATCACAACCC
	Reverse primer	GAAGGCACTGTCTCGGATGC
*Gapdh*	Forward primer	GGAGCGAGATCCCTCCAAAAT
	Reverse primer	GGCTGTTGTCATACTTCTCATGG

qRT-PCR = quantitative real-time polymerase chain reaction.

### 2.12. Colony formation assay

In each well of 6-well plates, 200 cells were cultivated in 2 mL of 1640 medium enriched with 10% FBS under typical settings for about 2 weeks to facilitate colony development. Subsequently, cells were immobilized with 1 mL of 4% paraformaldehyde (Solarbio, Beijing, China) for 15 minutes after being cleansed thrice with PBS. Thereafter, cells underwent triple PBS washes and were dyed with crystal violet (Beyotime) for 5 minutes. Colonies were tallied and scrutinized.

### 2.13. Transwell invasion assay

Invasion assays were performed using 24-well plates (Corning Inc., Shanghai, China). The lower chambers were filled with 600 μL of 1640 medium containing 10% FBS, and transwell inserts were placed in each well. A suspension of 8000 cells in 200 μL of medium was added to the upper chambers, and the cells were incubated for 24 hours. Nonmigrating cells were removed using a cotton swab, and the membranes were washed with PBS, fixed with 4% paraformaldehyde, and stained with crystal violet. Images of 5 random fields per insert were captured, and the number of invasive cells was counted.

### 2.14. Wound healing assay

Upon transfection, cells were seeded at a density of 3 × 10^5^ cells per well in 6-well plates and grown until they reached approximately 90% confluence. Using a 100 µL pipette tip, we created a scratch across the middle of each well. At 0, 24, 48, and 72 hours, we took pictures at 3 distinct points along each scratch to document wound dimensions and evaluate recovery rates among groups.

### 2.15. Statistical analysis

R software (version 4.4.1, Durham, NC) was used to conduct bioinformatic analyses. Statistical differences between the 2 groups were assessed using the Mann–Whitney *U* test, where a *P* value <.05 was considered statistically significant.

## 3. Results

### 3.1. Consensus clustering identifies 2 HNSCC molecular subtypes

**Figure 2.**
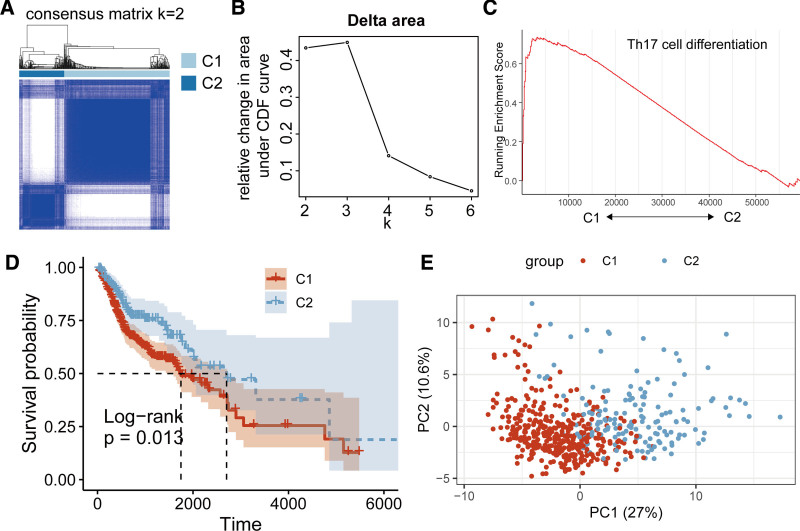
Molecular subtyping of HNSCC. (A, B) Consensus clustering analysis of the TCGA-HNSCC cohort based on 87 TCDRGs. (C) GSEA of the Th17 cell differentiation pathway across the 2 molecular subtypes (C1 and C2). (D) Kaplan–Meier survival analysis comparing the OS between subtypes C1 and C2. (E) PCA demonstrating the separation between the C1 and C2 subtypes. GSEA = gene set enrichment analysis, HNSCC = head and neck squamous cell carcinoma, OS = overall survival, PCA = principal component analysis, TCDRG = T-helper 17 cell differentiation–related genes, TCGA = The Cancer Genome Atlas.

We performed consensus clustering analysis on the TCGA-HNSCC cohort based on 87 TCDRGs (Fig. 2A and B), which resulted in the identification of 2 molecular subtypes (C1 and C2). GSEA revealed that the Th17 cell differentiation pathway was highly expressed in subtype C1 (Fig. 2C). Survival analysis revealed that patients in subtype C2 had a significantly better prognosis compared with those in C1 (*P* = .013; Fig. 2D). The principal component analysis demonstrated clear separation between the C1 and C2 subtypes (Fig. 2E). These findings suggest a strong association between Th17 cell differentiation and HNSCC prognosis, which could guide prognostic assessment.

### 3.2. Construction of a prognostic signature based on TCDRGs

**Figure 3.**
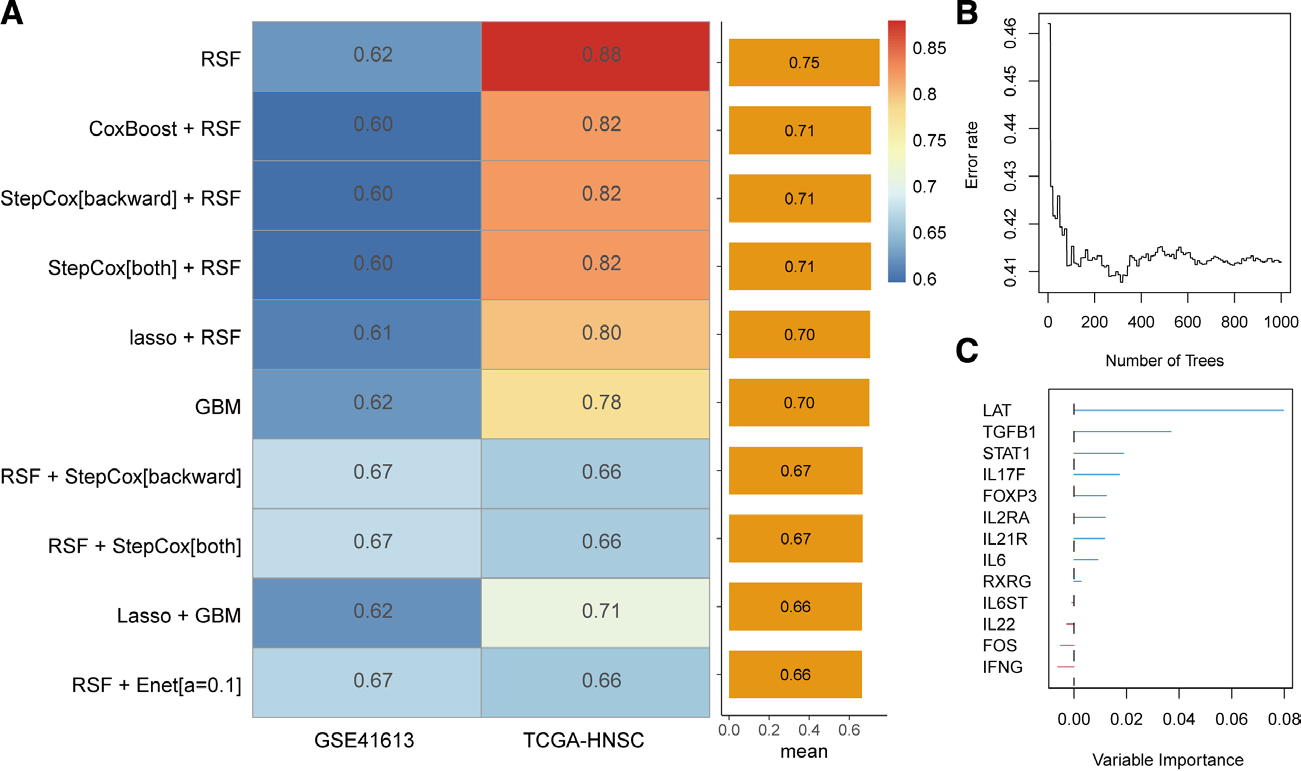
Construction of a prognostic signature based on machine learning algorithms. (A) Performance ranking of the top 10 machine learning algorithms. (B) Evolution of prediction error rates over 100 survival trees. (C) VIMP plot for the 13 selected TCDRGs. GBM = gradient boosting machine, RSF = random survival forest, TCDRG = T-helper 17 cell differentiation–related genes, TCGA = The Cancer Genome Atlas, VIMP = variable importance measure.

To create a prognostic model for patients with HNSCC using TCDRGs, we employed various machine learning algorithms and their combinations for feature selection and model construction. Through differential expression analysis, we identified 13 TCDRGs that were aberrantly expressed in HNSCC, with 7 genes (*TGFB1*, *IL2RA*, *FOXP3*, *STAT1*, *IFNG*, *IL21R*, and *LAT*) being significantly upregulated and 6 genes (*IL22*, *IL6ST*, *IL17F*, *FOS*, *RXRG*, and *IL6*) being significantly downregulated (Table S2, Supplemental Digital Content, http://links.lww.com/MD/O281). After applying 101 machine learning algorithms for feature selection and model building, we favored the random survival forest (RSF) model based on these 13 TCDRGs, as it had the highest average C-index among all tested models (Fig. 3A and Table S3, Supplemental Digital Content, http://links.lww.com/MD/O281). The evolution of 100 survival trees showed a trajectory of decreasing prediction error, confirming the robustness and reliability of the model (Fig. 3B). After the construction of 1000 trees, we derived variable importance measures (VIMP) for each feature during the tree formation process. Higher VIMP scores indicated a greater influence of genes on prognostic prediction. As shown in Figure 3C, the top 3 genes with the highest VIMP were *LAT*, *TGFB1*, and *STAT1*.

### 3.3. Evaluation of the RSF-based model

**Figure 4.**
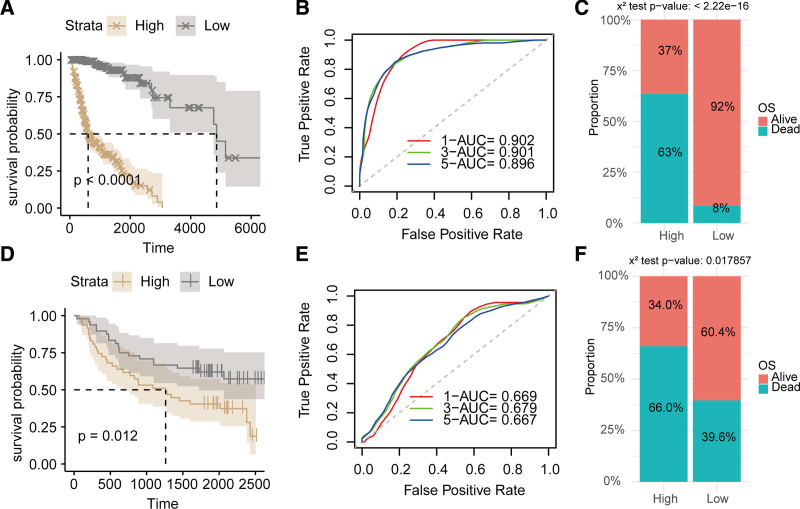
Performance evaluation of the TCDRG-based prognostic RSF model. (A) K–M survival analysis of the high- and low-risk groups in the TCGA-HNSCC cohort. (B) ROC curves for predicting 1-, 3-, and 5-year OS in the TCGA-HNSCC cohort. (C) Distribution of survival status between the high- and low-risk groups in the TCGA-HNSCC cohort. (D) K–M survival analysis of the high- and low-risk groups in the GSE41613 cohort. (E) ROC curves for predicting 1-, 3-, and 5-year OS in the GSE41613 cohort. (F) Distribution of survival status between the high- and low-risk groups in the GSE41613 cohort. AUC = area under the receiver operating characteristic curve, K–M = Kaplan–Meier, OS = overall survival, ROC = receiver operating characteristic, RSF = random survival forest, TCDRG = T-helper 17 cell differentiation–related genes, TCGA = The Cancer Genome Atlas.

Utilizing the RSF algorithm, we determined the risk index, T17I, for both the TCGA-HNSCC and GSE41613 datasets, categorizing participants into favorable and unfavorable risk strata according to the median threshold. Within the TCGA-HNSCC dataset, individuals within the favorable risk stratum exhibited substantially improved outcomes relative to their counterparts in the unfavorable risk stratum (*P* < .0001; Fig. 4A). The predictive efficacy for 1-, 3-, and 5-year overall survival (OS), as quantified by the AUC, reached 0.902, 0.901, and 0.896, respectively (Fig. 4B). Notably, the survival fraction among those in the favorable risk stratum (92%) vastly outperformed that observed in the unfavorable risk stratum (37%) (*P* < 2.22e−16; Fig. 4C). Analogously, within the GSE41613 dataset, subjects allocated to the favorable risk stratum also showcased superior outcomes vis-a-vis those in the unfavorable risk stratum (*P* = .012; Fig. 4D). The AUC metrics for forecasting 1-, 3-, and 5-year OS stood at 0.669, 0.679, and 0.667, correspondingly (Fig. 4E). Furthermore, the survival fraction within the favorable risk stratum (60.4%) was demonstrably elevated when juxtaposed against the unfavorable risk stratum (34.0%) (*P* = .017857; Fig. 4F). Collectively, these observations underscore the efficacy of the risk prediction model grounded in RSF and TCDRGs in accurately forecasting the clinical trajectory of patients with HNSCC.

### 3.4. Relationship between T17I and clinical characteristics

**Figure 5.**
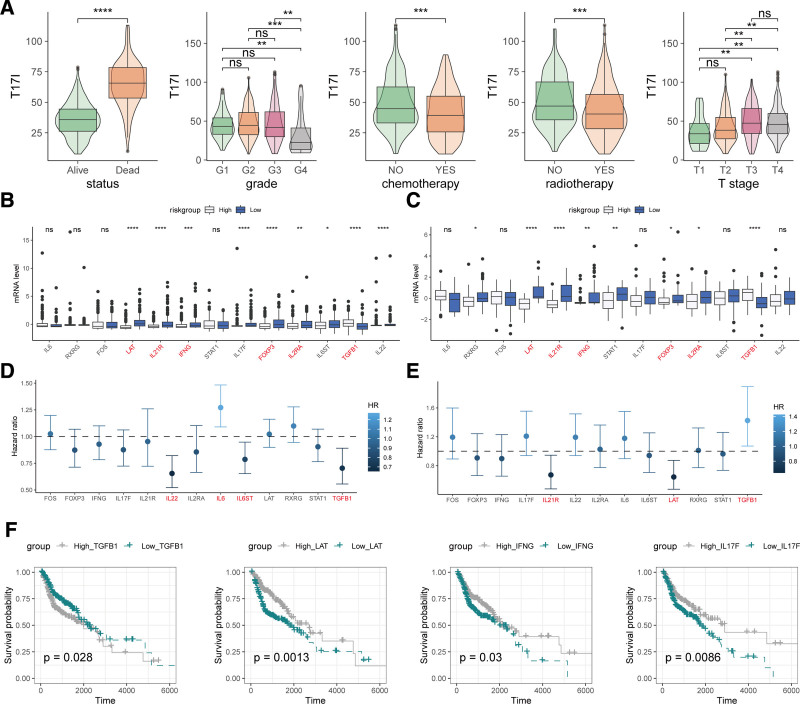
Relationship between T17I and clinical–pathological features, and expression and prognostic analysis of related genes. (A) Comparison of T17I across different clinical–pathological subgroups. (B) Differential expression of T17I-related genes between the high- and low-risk groups in the TCGA-HNSCC cohort. (C) Differential expression of T17I-related genes between the high- and low-risk groups in the GSE41613 cohort. (D) Hazard ratios of T17I-related genes in the TCGA-HNSCC cohort. (E) Hazard ratios of T17I-related genes in the GSE41613 cohort. (F) K–M survival analysis of T17I-related genes divided into high- and low-expression groups based on median values in the TCGA-HNSCC cohort. *P < .05, **P < .01, ***P < .001, ****P < .0001. K–M = Kaplan–Meier, ns = not significant, TCGA = The Cancer Genome Atlas.

We evaluated T17I across different clinical–pathological subgroups and found that T17I was associated with survival outcome, grade, chemotherapy/radiotherapy events, and T stage. Specifically, deceased patients had higher T17I compared with alive patients, higher-grade tumors had lower T17I compared with lower-grade tumors, advanced T stage had higher T17I compared with early T stage, and patients who received chemotherapy/radiotherapy had higher T17I compared with those who did not (Fig. 5A). In contrast to the high-risk category, the low-risk category displayed notably enhanced expression levels of LAT, IL21R, IFNG, FOXP3, and IL2RA, alongside significantly reduced expression of TGFB1 across both the TCGA-HNSCC and GSE41613 cohorts (Fig. 5B and C). Figure 5D and E illustrates hazard ratios associated with genes linked to T17I in the respective TCGA-HNSCC and GSE41613 datasets. Within the TCGA-HNSCC cohort, patients were categorized into high- and low-expression groups based on the median expression level of each gene, followed by K–M analysis. The findings revealed that diminished expression of TGFB1 and heightened expression of LAT, IFNG, and IL17F were significantly correlated with an improved prognosis in patients with HNSCC (Fig. 5F).

### 3.5. Relationship between T17I and somatic mutations

**Figure 6.**
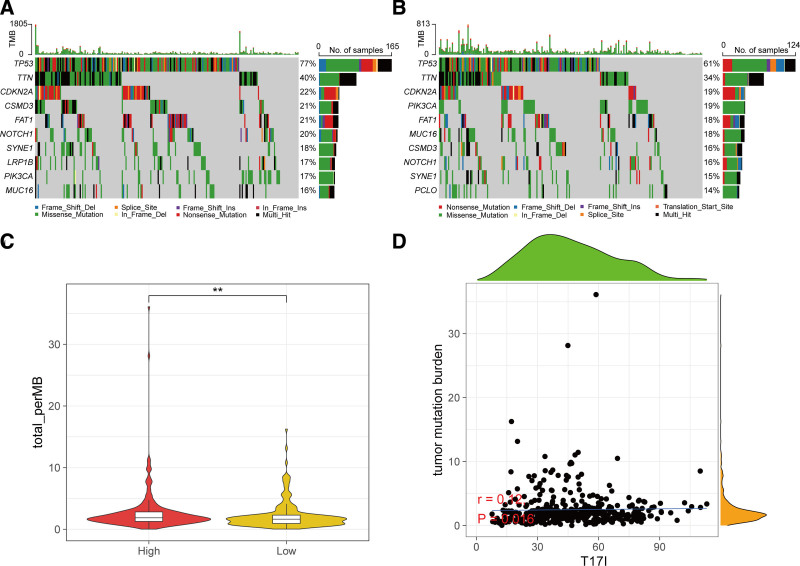
Analysis of the correlation between T17I and somatic mutations. (A) Oncoplot displaying the top 10 most frequently mutated genes in the high-risk group. (B) Oncoplot displaying the top 10 most frequently mutated genes in the low-risk group. (C) Comparison of TMB between the high- and low-risk groups. (D) Scatter plot and correlation analysis between TMB and T17I. **P < .01. TMB = tumor mutational burden.

To delve deeper into the association between T17I and somatic mutations, we examined the top 10 most frequently mutated genes. Our investigation disclosed that the leading mutated genes in the high-risk category included *TP53*, *TTN*, *CDKN2A*, *CSMD3*, *FAT1*, *NOTCH1*, *SYNE1*, *LRP1B*, *PIK3CA*, and *MUC16*, whereas in the low-risk category, they comprised *TP53*, *TTN*, *CDKN2A*, *PIK3CA*, *FAT1*, *MUC16*, *CSMD3*, *NOTCH1*, *SYNE1*, and *PCLO* (Fig. 6A and B). When comparing the low-risk group to the high-risk group, we noted a considerably greater TMB in the latter (Fig. 6C). Statistical correlation analysis unveiled a modest yet statistically significant positive relationship (*r* =  0.12, *P* = .0016) between T17I and TMB (Fig. 6D).

### 3.6. Gene expression patterns associated with T17I

**Figure 7.**
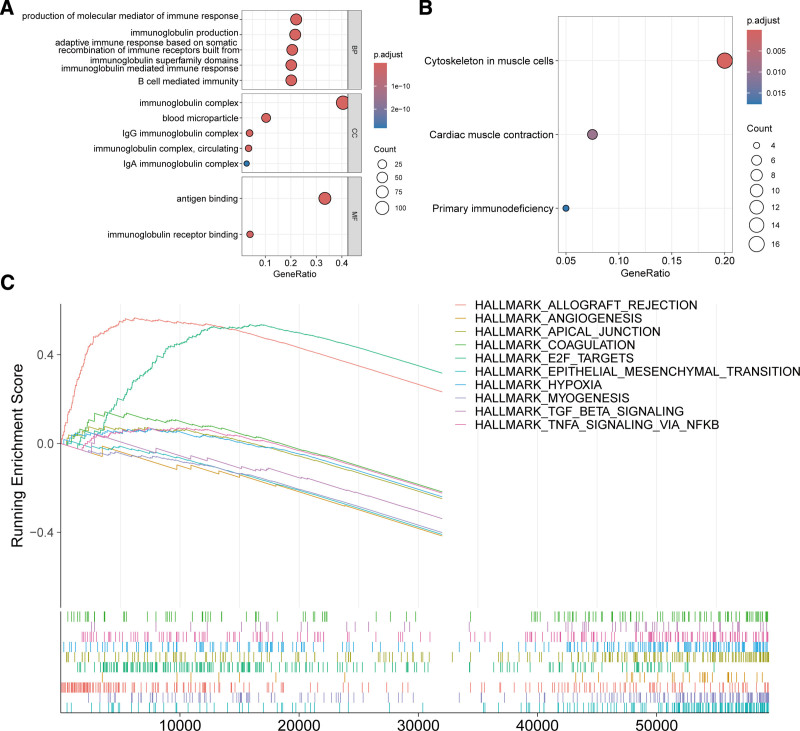
Analysis of the underlying mechanisms linking T17I and prognosis. (A) GO enrichment analysis of differentially expressed genes between the high- and low-risk groups. (B) KEGG pathway enrichment analysis of differentially expressed genes between the high- and low-risk groups. (C) GSEA of significantly differentially enriched gene sets between the high- and low-risk groups. GO = Gene Ontology, GSEA = gene set enrichment analysis, KEGG = Kyoto Encyclopedia of Genes and Genomes.

To further investigate the underlying mechanisms related to T17I and its impact on prognosis, we conducted an analysis of differentially expressed genes between 2 risk groups, resulting in the identification of 377 distinct genes (Table S4, Supplemental Digital Content, http://links.lww.com/MD/O281). GO enrichment analysis indicated that these genes were involved in immune response, B-cell-mediated immunity, antigen, and immunoglobulin receptor binding (Fig. 7A). KEGG pathway enrichment analysis showed that these genes were involved in pathways such as cytoskeleton in muscle cells, cardiac muscle contraction, and primary immunodeficiency (Fig. 7B). GSEA uncovered 19 gene sets that were significantly enriched in either the high- or low-risk categories (Table S5, Supplemental Digital Content, http://links.lww.com/MD/O281 and Fig. 7C), including allograft rejection, E2F targets, and G2M checkpoint in the high-risk group, and epithelial–mesenchymal transition, myogenesis, apical junction, TGF-β signaling, tumor necrosis factor alpha signaling, hypoxia, and angiogenesis in the low-risk group. Collectively, these data shed light on the potential mechanisms linking T17I and HNSCC prognosis.

### 3.7. Relationship between T17I and the tumor immune landscape

**Figure 8.**
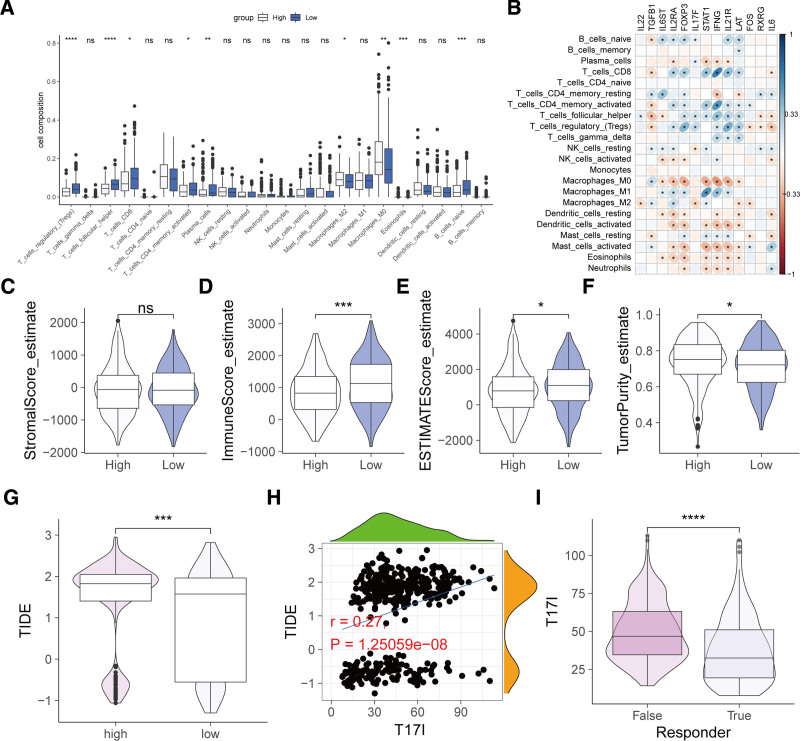
Correlation between T17I and the tumor immune microenvironment. (A) Comparison of immune cell infiltration between the high- and low-risk groups. (B) Heat map illustrating the correlation between T17I-related genes and immune cell infiltration. (C–F) Assessment of immune scores and tumor purity in the high- and low-risk groups. (G) Comparison of TIDE scores between the high- and low-risk groups. (H) Scatter plot and correlation analysis between TIDE and T17I. (I) Comparison of T17I between true and false responders to immunotherapy. *P < .05, **P < .01, ***P < .001, ****P < .0001. ns = not significant, TIDE = tumor immune dysfunction and exclusion.

Next, we assessed the immunoinfiltration within the TCGA-HNSCC cohort. The results demonstrated that in comparison with the high-risk group, the low-risk group exhibited substantially higher infiltration levels of regulatory T cells, follicular helper T cells, CD8^+^ T cells, activated CD4^+^ memory T cells, plasma cells, and naive B cells; in contrast, the infiltration levels of M2 and M0 macrophages were considerably lower (Fig. 8A). There were complex and significant correlations between T17I-related genes and immune cell infiltration, with most genes positively correlated with T cells and negatively correlated with macrophages, dendritic cells, mast cells, eosinophils, and neutrophils (Fig. 8B). The ESTIMATE algorithm–based analysis revealed that, in contrast to the high-risk group, the low-risk group possessed a higher immune score and lower tumor purity (Fig. 8C–F). These findings indicate that the high-risk group has a more immunosuppressive microenvironment. In addition, compared with the high-risk group, the low-risk group had lower TIDE scores (Fig. 8G), and TIDE was positively correlated with T17I (Fig. 8H). Compared with false responders, true responders to immunotherapy had lower T17I (Fig. 8I). These results indicate that lower T17I is associated with higher responsiveness to immunotherapy.

### 3.8. Association between T17I and chemotherapy sensitivity in HNSCC

**Figure 9.**
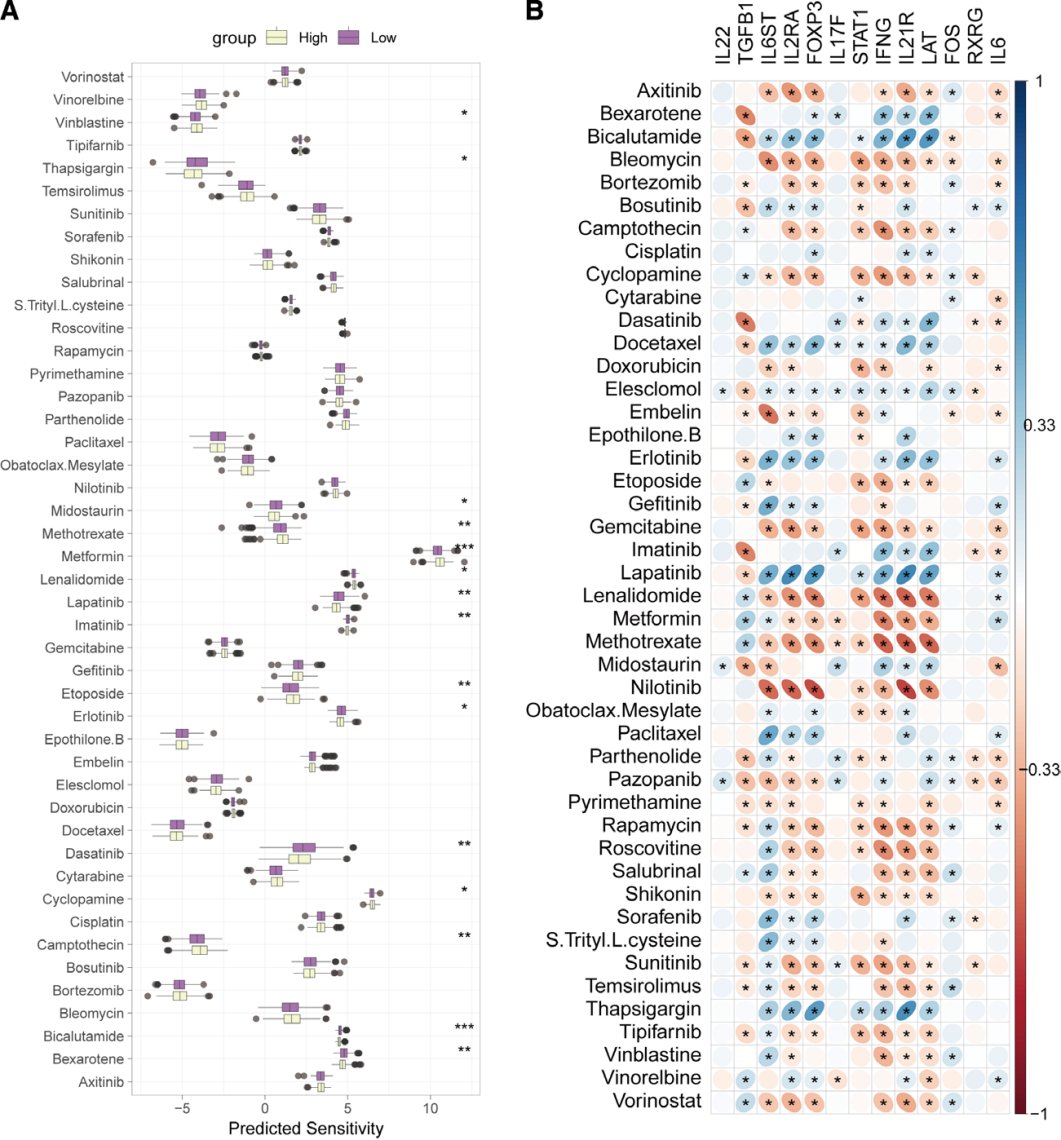
Correlation between T17I and drug sensitivity. (A) Comparison of drug sensitivity between the high- and low-risk groups for 45 drugs. (B) Heat map illustrating the correlation between T17I-related genes and drug sensitivity. *P < .05, **P < .01, ***P < .001.

Chemotherapy is a key component of cancer treatment, so we evaluated the sensitivity of the TCGA-HNSCC cohort to 45 drugs. The results showed that the low-risk group had higher sensitivity to vinorelbine, metformin, lenalidomide, etoposide, cyclopamine, and camptothecin and lower sensitivity to thapsigargin, midostaurin, lapatinib, imatinib, erlotinib, dasatinib, bicalutamide, and bexarotene compared with the high-risk group (Fig. 9A). Correlation analysis demonstrated complex relationships between T17I-related genes and drug sensitivity, with IL6ST, IL2RA, FOXP3, IFNG, IL21R, and *LAT* showing consistent sensitivity patterns, whereas TGFB1 had opposite sensitivity patterns and IL22 had lower correlation with drug sensitivity (Fig. 9B).

### 3.9. Development of a nomogram based on T17I

**Figure 10.**
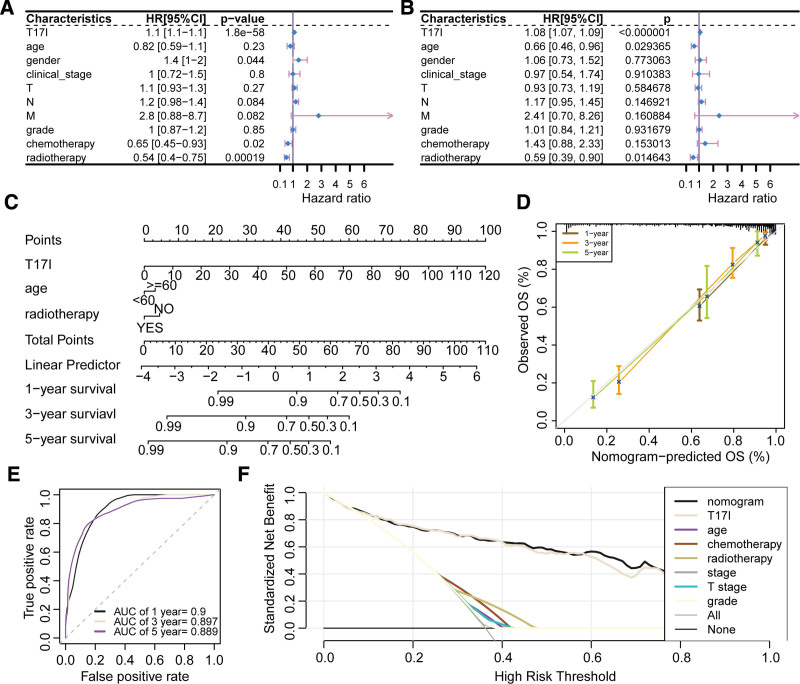
Development and evaluation of a nomogram. (A) Univariate Cox regression analysis of prognostic factors. (B) Multivariate Cox regression analysis of independent prognostic factors. (C) Nomogram incorporating T17I, age, and radiotherapy to predict 1-, 3-, and 5-year overall survival. (D) Calibration curve analysis of the nomogram model. (E) ROC curve analysis of the nomogram model. (F) Decision curve analysis of the nomogram model. AUC = area under the receiver operating characteristic curve, CI = confidence interval, HR = hazard ratio, OS = overall survival, ROC = receiver operating characteristic.

Univariate Cox regression analysis suggested that T17I, gender, chemotherapy, and radiotherapy were factors related to the prognosis of HNSCC (Fig. 10A). T17I, age, and radiotherapy were determined as independent prognostic factors through multivariate Cox regression analysis (Fig. 10B). These factors were utilized to build a nomogram model for predicting 1-, 3-, and 5-year OS (Fig. 10C). The calibration curves indicated a good consistency between the OS predicted by the nomogram and the actual OS (Fig. 10D). It was demonstrated that the nomogram attained AUC values of 0.9, 0.897, and 0.889 for forecasting 1-, 3-, and 5-year OS outcome, correspondingly (Fig. 10E). These results indicate excellent performance of the nomogram in prognostic assessment. Furthermore, decision curve analysis showed that the nomogram had a higher standardized net benefit compared with other factors for predicting 1-year OS (Fig. 10F), suggesting superior predictive performance.

### 3.10. Biological functions of *LAT* in HNSCC

**Figure 11.**
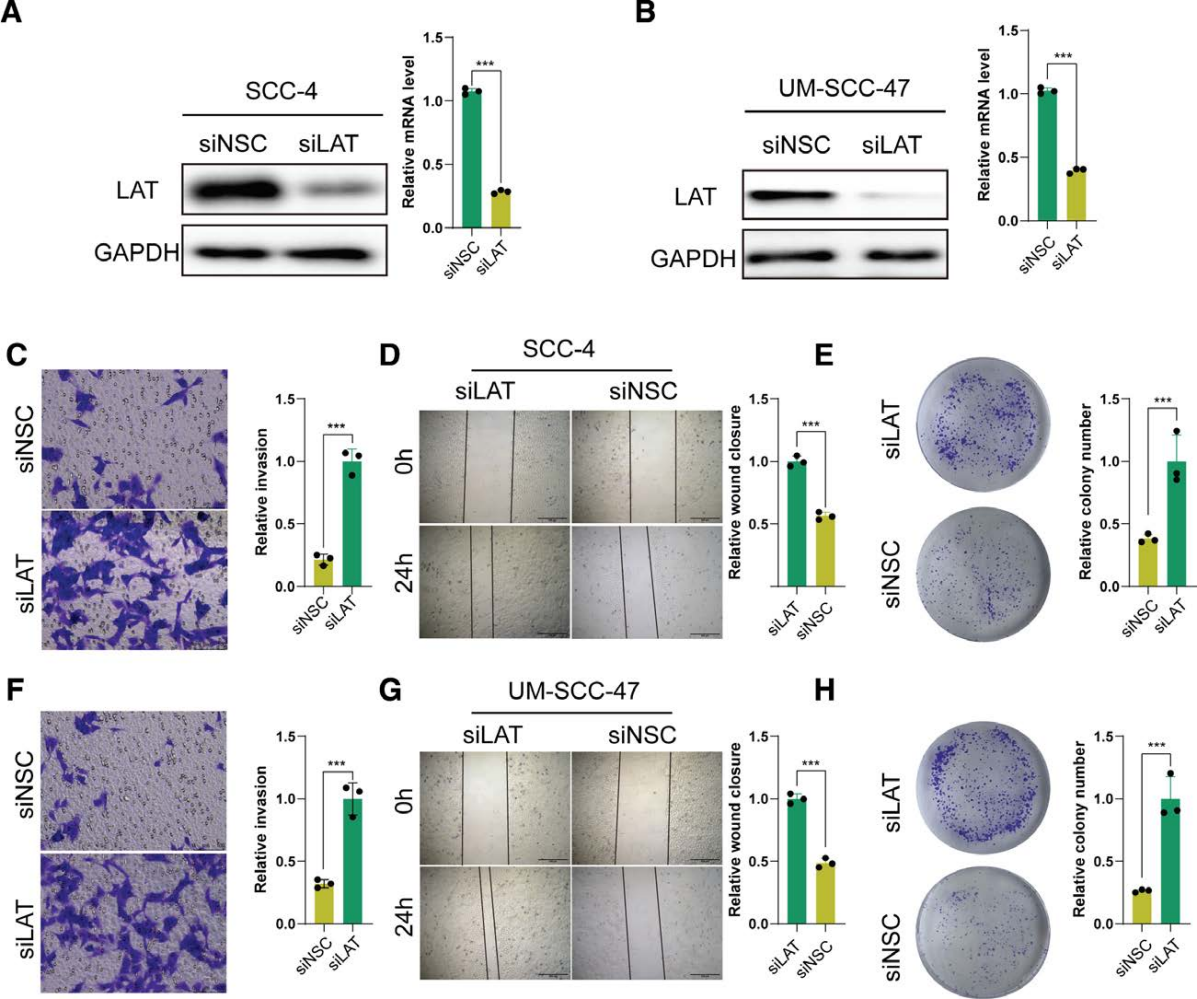
Biological function of *LAT* in HNSCC. Western blot and RT-PCR analysis of SCC-4 (A) or UM-SCC-47 (B) cells after transfection with LAT-targeting siRNAs or siNSC. (C–E) Effects of *LAT* on invasion, migration, and proliferation of SCC-4 cells assessed by transwell assays (scale bars = 100 μm), Scratch assays (scale bars = 500 μm), and colony formation assays. (F–H) Effects of *LAT* on invasion, migration, and proliferation of UM-SCC-47 cells assessed by transwell assays (scale bars = 100 μm), scratch assays (scale bars = 500 μm), and colony formation assays. Bar charts represent mean ± SD. ***P < .001. HNSCC = head and neck squamous cell carcinoma, RT-PCR = real-time polymerase chain reaction, siNSC = nonsilencing control siRNA, siRNA = small interfering RNA.

Based on VIMP, we selected *LAT* for functional validation. To determine the cellular function of LAT, we chose 2 different HNSCC cancer cell lines, SCC-4 and UM-SCC-47, for in vitro knockdown experiments. Then, we transfected these cell lines with either LAT-targeted siRNA or nonsilencing control siNSC, confirming the downregulation of gene expression via western blotting and RT-PCR analysis (Fig. 11A and B). The transwell invasion assay demonstrated a significant enhancement in the relative frequency of invasive cells within the siLAT group compared with the siNSC group (Fig. 11C and F). Scratch assays revealed that *LAT* silencing substantially accelerated wound healing in SCC-4 and UM-SCC-47 cells (Fig. 11D and G). Furthermore, the colony formation assay showcased a marked rise in colony numbers after *LAT* suppression in both SCC-4 and UM-SCC-47 cells (Fig. 11E and H). These outcomes propose that *LAT* inhibition fosters cellular proliferation, motility, and invasiveness in HNSCC cells. Taken together, these investigations underscore the pivotal function of TCDRGs, specifically LAT, in HNSCC advancement and point toward promising therapeutic avenues for intervention.

## 4. Discussion

HNSCC represents a significant health concern due to its suboptimal clinical outcomes, primarily attributed to local recurrence and distant metastasis.^[19]^ Consequently, identifying novel predictive biomarkers is important for achieving personalized treatment strategies for HNSCC.^[20,21]^ Herein, we constructed an original prognostic signature on the basis of TCDRGs with the TCGA training cohort and verified its stability through both internal TCGA validation and external Gene Expression Omnibus cohorts. By employing the RSF algorithm, a risk signature (T17I) was computed to predict the patient’s prognosis. The analysis demonstrated that individuals categorized in the high-risk group exhibited notably reduced OS compared with those in the low-risk group, across both the training and validation cohorts. The predictive accuracy of the 13 TCDRG-based signature for survival was further substantiated by ROC curves in both cohorts. These results indicated that the T171 serves as a robust tool for evaluating the prognosis of patients with HNSCC.

Th17 cells and associated cytokines have been shown to both promote and suppress tumor growth, although the exact mechanisms remain unclear.^[11]^ Our study demonstrated that TCDRGs could stratify HNSCC into 2 subtypes with distinct prognoses, where the subtype with poorer prognosis exhibited high expression of TCDRGs, indicating a link between Th17 cell activity and HNSCC progression. Earlier research has indicated that TH17 cells occur more frequently in patients with HNSCC and that their levels are associated with the TNM staging of the tumor, implying their involvement in tumor progression and spread.^[22]^ These cells can be generated and proliferate within the TME due to cytokines secreted by both tumor cells and tumor-infiltrating lymphocytes. In addition, they can be attracted to the tumor site through mechanisms dependent on CCR6/CCL20.^[23]^ In addition, the presence of Th17 cells has been shown to inhibit proliferation and angiogenesis in HNSCC.^[23]^ Further investigation into the mechanisms of Th17 cell differentiation and recruitment in HNSCC will help elucidate their role in promoting or inhibiting tumor progression.

Our analysis revealed that the 13 TCDRGs associated with T17I were dysregulated in HNSCC, suggesting that TCDRGs may play a significant role in cancer development and progression. Recent studies have implicated these TCDRGs in tumor initiation and progression. For example, TGFB1 induces THBS1, a tumor-specific extracellular matrix protein, partially through integrin signaling, promoting cancer cell migration, and stimulating matrix metalloproteinases expression, thereby facilitating oral squamous cell carcinoma invasion.^[24]^ Studies have shown that IL2RA promotes the invasiveness and stem cell-related characteristics of acute myeloid leukemia.^[25]^ In addition, IL2RA serves as a prognostic indicator in pancreatic ductal adenocarcinoma.^[26]^ FOXP3 is highly expressed in HNSCC,^[27]^ and its expression varies by anatomical site and correlates with tumor stage and poor prognosis.^[28,29]^ Consequently, the expression of Foxp3 in primary tumors and lymph node metastases may identify patients at high risk for HNSCC.^[28]^ Tumor-infiltrating FoxP3-positive T cells have been widely recognized as poor prognostic factors in HNSCC.^[30–33]^ STAT1, which is regulated by TRIM24, contributes to immunosuppression in HNSCC cells but prevents T-cell exhaustion and the buildup of myeloid-derived suppressor cells in the TME, thereby boosting T-cell antitumor immunity.^[34,35]^ IFNG is critical for immune regulation and influences the TME and related signaling pathways, such as Janus Kinase-Signal Transducer and Activator of Transcription and Nuclear Factor Kappa B.^[36,37]^ The expression levels of IFNG can serve as biomarkers for certain cancers, aiding in the assessment of patient prognosis and treatment response.^[38]^ Recent studies have shown that low methylation of IL21R can serve as a biomarker to distinguish between benign and malignant breast tumors.^[39]^

*LAT* is a critical signaling protein primarily expressed in T cells. It is responsible for transmitting signals from cell surface receptors to the intracellular environment, thereby regulating T-cell activation and function. Latent dysfunction of *LAT* may hinder the immune system’s capacity to detect and destroy tumor cells.^[40]^ This observation aligns with our in vitro findings, which indicate that reducing *LAT* gene expression enhances the proliferation, metastasis, and invasion of HNSCC cells. IL22 can stimulate the activation and growth of immune cells by activating the STAT3 pathway.^[41]^ IL6ST acts as a prognostic indicator in breast cancer^[42]^ and could potentially serve as a therapeutic target in colon cancer by modulating ferroptosis.^[43]^ Variations in the *IL17F* gene are frequently linked to cancer risk.^[44]^ RXRG serves as an independent prognostic marker in estrogen receptor–positive breast cancer.^[45]^ Elevated expression of c-fos in oral squamous cell carcinoma facilitates cell invasion and migration via the CD44 pathway.^[46]^ In the HNSCC TME, FOS exhibits differential expression across various cell populations, particularly in fibroblasts and myeloid cells.^[47]^ This varied expression likely indicates the different roles these cells play in tumor development and their influence on the TME. In addition, c-Fos functions as a modulator of epithelial–mesenchymal transition and cancer stem cell reprogramming in HNSCC cells.^[48]^ IL-6, a versatile cytokine, enhances tumor cell proliferation and metastasis by activating the STAT3 signaling pathway.^[49]^

To investigate the biological roles of TCDRGs, we conducted an enrichment analysis. Notably, the results revealed significant enrichment of key immune-related processes and pathways, including immune response, B-cell-mediated immunity, antigen, and immunoglobulin receptor binding. Modulation of the TME by the immune system is crucial for the development and progression of HNSCC.^[50,51]^ Based on our findings, TCDRGs might impact HNSCC metastasis, angiogenesis, or tumor growth through mechanisms involving the immune system.

Our study indicates that the TCDRG-derived risk signature correlates with the tumor immune microenvironment, marked by a notable increase in B-cell and T-cell infiltration in patients with risk scores. The infiltration of these immune cells is conducive to generating an effective antitumor immune response, which can explain why patients with a lower risk score have a better prognosis.^[52,53]^ In addition, M2 macrophages possess protumorigenic activity, contributing to TME remodeling, angiogenesis, tissue repair, and immunosuppression.^[54]^ They release cytokines such as IL-10 and TGF-β, which inhibit immune responses and facilitate tumor cell survival and progression.^[55]^ Thus, the positive correlation between T17I and M2 macrophage infiltration may be related to its role in guiding prognosis. Further investigation is warranted to elucidate the relationship and underlying mechanisms between T17I-associated genes and M2 macrophage infiltration.

Our study has several limitations. First, the use of a retrospective cohort for model construction and validation may introduce selection bias and information bias, potentially affecting the stability and predictive power of the model. Additional large-scale prospective clinical trials are necessary to validate our findings and evaluate the practical clinical utility of this prognostic model. Second, although we identified a set of TCDRGs, the precise roles of these genes in Th17 cell differentiation and their biological functions in HNSCC remain unclear. Future research should delve deeper into the mechanisms of action of these genes and their roles in the TME, which is crucial for understanding the role of Th17 cells in HNSCC progression and for developing new therapies. Finally, although our study demonstrated a link between TCDRGs and HNSCC prognosis, the distinct expression profiles of these genes across various HNSCC subtypes and their associations with clinical characteristics warrant additional in-depth investigation. Future research should focus on the expression differences of these genes in specific subtypes and investigate whether they can serve as biomarkers for personalized treatment strategies in HNSCC.

## 5. Conclusion

In summary, our study developed a molecular classification and prognostic signature based on TCDRGs for HNSCC, identified 2 subtypes with distinct prognoses, and constructed a risk score model consisting of 13 TCDRGs. We further constructed a nomogram that integrates the risk score, patient age, and radiotherapy status to aid in personalized treatment planning for HNSCC. Nevertheless, additional research is required to clarify the molecular mechanisms governing the role of TCDRGs in Th17 cell differentiation and their contribution to HNSCC progression.

## Acknowledgments

The authors thank Dr Yanbi Dai (Department of Otolaryngology, The First People’s Hospital of Yuhang District, Hangzhou) for her invaluable assistance with the data acquisition and bioinformatic analysis.

## Author contributions

**Data curation:** Shiqin Chen, Pingcun Wei, Gang Wang, Fan Wu, Jianjun Zou.

**Formal analysis:** Shiqin Chen, Pingcun Wei, Jianjun Zou.

**Investigation:** Shiqin Chen, Pingcun Wei, Gang Wang, Fan Wu, Jianjun Zou.

**Resources:** Shiqin Chen, Jianjun Zou.

**Software:** Shiqin Chen, Jianjun Zou.

**Validation:** Shiqin Chen, Pingcun Wei, Gang Wang, Fan Wu, Jianjun Zou.

**Visualization:** Shiqin Chen, Pingcun Wei, Gang Wang, Fan Wu, Jianjun Zou.

**Writing – review & editing:** Shiqin Chen, Jianjun Zou.

**Conceptualization:** Jianjun Zou.

**Funding acquisition:** Jianjun Zou.

**Methodology:** Jianjun Zou.

**Project administration:** Jianjun Zou.

**Supervision:** Jianjun Zou.

**Writing – original draft:** Jianjun Zou.

## Supplementary Material


